# Vascularised Composite Allotransplantation – A guide to optimal dissemination of scientific outputs

**DOI:** 10.1016/j.jham.2024.100161

**Published:** 2024-09-21

**Authors:** C.M. Hehir, G.P. Dowling, G.G. Calpin, M. O'Connor, L. Kelly, C.S. Honeyman, H.L. Stark, R.T. Dolan

**Affiliations:** a Department of Surgery, RCSI University of Medicine and Health Sciences, Dublin, Ireland; b Tissue Engineering Research Group (TERG), Department of Anatomy and Regenerative Medicine, RCSI University of Medicine and Health Sciences, Dublin, Ireland; c Department of Plastic & Reconstructive Surgery, Beaumont Hospital, Dublin, Ireland; d School of Medicine, Royal College of Surgeons in Ireland, Dublin, Ireland; e Department of Plastic Surgery, Derriford Hospital, Plymouth, UK; f Transplant Research and Immunology Group (TRIG), Nuffield Department of Surgical Science, University of Oxford, Oxford, UK

**Keywords:** Vascularised composite allograft, Transplant, Altmetric analysis, Sentinel skin flap

## Abstract

**Purpose:**

The impact of academic research is not just important within the clinical domain but within society as a whole. Altmetric Attention Score (AAS) offers a means of assessing how scholarly outputs are interacted with online. Vascularised Composite Allotransplantation (VCA) is a modern but rapidly evolving topic which encompasses a broad range of complex and clinically significant surgical interventions. Primarily VCA is utilised in the reconstruction of complex, composite tissue defects, including limb and face transplantation. There is also a growing interest in the role of VCA as an early means of real-time immuno-monitoring, in sentinel skin flap transplant (SSF).

**Materials & methods:**

In July 2024, a search was conducted using the *Altmetric Explorer (AE)* database using the search term ‘vascular-composite’ AND ‘allograft’ OR ‘allotransplant’. A simultaneous literature search was carried out using Web of Science (WoS) database utilising the same search terms with extraction of traditional citation-based metrics as well as relevant ‘*meso’* and ‘*micro’* subject headings. Corresponding citation-based metrics were extracted utilising *SCImago*. Data was compiled and analysed using a linear regression model with level of significance set at p < 0.05.

**Results:**

The Top 100 (T100) performing articles relating to VCA displayed a mean Altmetric Attention Score (AAS) of 3.31. All T100 papers were published in the English language. Sixty percent (n = 60) of T100 papers were published in Q1 Journals. News outlet mentions (r = 6.95), blog mentions (r = 6.20), and *Twitter/X* mentions (r = 0.52) demonstrated the greatest positive impact on AAS upon application of a linear regression model (p < 0.05).

**Conclusion:**

Altmetric Scores offer a means of appraising the impact of research outputs in both academic and societal domains. Such modern metrics are useful in evolving topics such as VCA as AAS is not dependent on citation counts. Publishing of outputs in high quartile, open access journals with timely utilisation of news and social media outlets should be utilised by researchers aiming to maximise dissemination of research outputs in the field of VCA.

## Highlights

• Vascularised Composite Allotransplantation (VCA) is a rapidly advancing surgical intervention aimed at treatment of complex tissue deficits.
• Rising numbers of upper limb, facial, abdominal wall, uterine and penile VCAs are being performed globally.
• Research publications on the topic of VCA have demonstrated steady growth over the past decade, as has online attention on the topic.
• Altmetrics offers a means of appraising the level of interaction surrounding scholarly outputs, with implications for optimal dissemination.

## 1 Introduction

Vascularised composite allotransplantation (VCA) involves the transfer of multiple tissue types from donor to recipient as a single functional unit.^1^ This therapeutic modality has revolutionised the treatment of complex tissue deficits, providing reconstructive opportunities to patients who have suffered detrimental tissue loss. The field of VCA has evolved significantly since the first reported case of hand transplantation in 1964, thanks to significant advances in immunosuppressive therapies and microsurgical expertise.^2^^,^^3^ The application of VCA is ever-growing globally with successful upper extremity, craniofacial, abdominal wall, penis and uterus transplantation being reported in increasing numbers.^4^, ^5^, ^6^, ^7^

There is a smaller, but evolving application of VCA known as Sentinel Skin Flap transplantation, in which a VCA, composed of skin and subcutaneous tissue, is transplanted simultaneously alongside a solid organ transplant (SOT).^8^ The concept of SSF transplantation relies on the favourable immunogenicity profile of cutaneous tissue and ease in visualisation. As such, SSF provides a dynamic canvas capable of identification of rejection episodes in a transplanted visceral organ, allowing for real-time immune-monitoring and early detection of visible rejection episodes, prior to clinical signs of rejection within the SOT.^9^, ^10^, ^11^ There is growing evidence that mucosal components of VCA may act as an equal, if not superior, canvas in early identification of rejection from both macro and microscopic perspectives.^12^^,^^13^ The increase in acceptability of VCA as a treatment modality with regards to both VCA donation and implantation is being increasingly reported globally.^14^^,^^15^ Further research is ongoing aimed at ensuring the growing application of VCA is carried out in a manner acceptable to donors, recipients and the general public.^16^

The evolution of social media and other online interfaces has perpetually transformed the means in which academic outputs are both interacted with and disseminated.^17^ Alternative metrics – *Altmetrics* – have emerged has a modern means of measuring the degree and type of online interaction generated by research outputs.^18^^,^^19^ Altmetrics generate interaction data from social media outlets, major news outlets, public policy documentations and reference managers such as Mendeley, amongst others.^18^ This means of assessing research interaction complements that of traditional citation-based metrics whilst capturing a broader societal impact, detailing interaction both within and outside the academic community.^20^ Altmetric Attention Score (AAS) are generated more rapidly than citation-based metrics, which may be preferrable for appraisal in evolving and niche topics such as VCA.^18^ Altmetrics have been implemented across a broad range of topics, with journals utilising these statistics to generate targeted approaches to increasing interaction with their publications.^21^^,^^22^ Recent studies have reinforced the quality of altmetric data and its resistance to manipulation as analysed using Benford's Law.^23^

This study aims to appraise the current top performing articles relating to VCA research from an Altmetrics perspective. We aim to identify the factors which aid broad and timely dissemination of outputs and build a comprehensive guide to provide guidance for researchers aiming to publish in this evolving field.

## 2 Materials & Methods

In July 2024, a search was conducted using the *Altmetric Explorer* (AE) database (Altmetric LLP, London, UK) using the search terms ‘vascular-composite’ AND ‘allograft’ OR ‘allotransplant’. The top 100 (T100) outputs were generated using AAS and exported for analysis. Representative graphical representations of Altmetric datasets were extracted from AE and included as figures. *SCIimago Country and Journal Rank* was utilised to generate journal quartile, impact factor, H-index and SJR. T100 outputs were cross-referenced using the Web of Science (WoS) database, ‘*Meso’* and ‘*Micro’* subject headings were manually extracted and inputted into *Microsoft Excel* for analysis. A separate search was carried out using WoS with the search terms ‘vascular-composite’ AND ‘allograft’ OR ‘allotransplant’, with outputs extracted for analysis. The WoS ‘analyse results’ function was used to generate graphs representative of research outputs over time. Descriptive and correlational statistics were generated using *Microsoft Excel and STATA* using linear regression models. A threshold for significance was set at p < 0.05.

## 3 Results

The T100 journal articles displayed a mean AAS of 3.31 (Range 0–60). All T100 papers were written in the English language with 63 of T100 papers being published in American journals. The United Kingdom (n = 21), Italy (n = 7), Switzerland (n = 4), New Zealand (n = 1), the Netherlands (n = 1), China (n = 1), Egypt (n = 1) and Germany (n = 1) also contributed to T100 publications. The majority of T100 papers were published in Q1 Journals (n = 60), with mean AAS being significantly higher within Q1 publications [4.18] in comparison to Q2[2.18] and Q3^1^. There was a dominance of Open Access (OA) Journal publications (n = 64) with 7 of the top 10 performing articles being published as OA [Table 1].

**Table 1:** Frequency and Descriptive statistics of included T100 journals reporting on SCImago Journal Ranking, Article Access, Study Type and Journal Presence of social media.

Factor	Number of Outputs (Total = 100)	Mean AAS (min-max)
SCImago Journal Ranking		
Q1	60	4.18(0–60)
Q2	34	2.18(0–12)
Q3	5	1(0–2)
Not Indexed	1	13(−)
Article Access		
Open Access	64	64 %
Paid Access	36	36 %
Study Type		
Review	25	1.72(0–7)
Cohort	8	2(0–6)
Experimental	55	4.47(0–60)
	In Vivo = 46 Ex Vivo = 9	
Case Report	2	1(1–1)
Case Series	2	6(0–12)
Qualitative Analysis	4	3.5(1–6)
Non-scholarly Outputs	4	0.75(0–2)
Journal Presence on Social Media		
Yes	86	3.66 (0–60)
No	14	1.5 (0–12)

Publication in journals which had a dedicated social media page generated higher mean AAS (3.66 [range: 0–60]) in comparison to those who did not (1.5 [range:0–12]). The majority of T100 publications (n = 67) have been published in the past 5 years. Linear regression analysis did not demonstrate any statistically significant relationship between AAS and Journal Quartile.

The majority of T100 publications were interventional animal studies (n = 53), with murine VCA transplantation being the most common animal VCA study subtype (n = 27). Swine, Non-Human Primates and Canine subjects were also investigated. Human VCA contributed n = 10 of the T100 studies with Facial VCA transplantation being the most frequent subtype (n = 4) followed by mixed VCA (n = 3), Upper Limb VCA (n = 2) and SSF VCA (n = 1). Articles which did not involve surgical intervention were mostly centred around factors affecting VCA procurement, allocation or implementation (n = 15) and VCA immunology (n = 15), [Table 2].

**Table 2:** Outline of T100 outputs in accordance study design.

VCA- Preclinical Models		
Animal (n = 53)	Murine	27
	Swine	17
	Non-Human Primates	7
	Canine	2
Cadaveric (n = 4)	Bladder	2
	Abdominal	1
	Mixed VCA	1
**VCA - Clinical Models**		
Human (n = 10)	Facial	4
	Mixed Transplant	3
	Upper Limb	2
	VCA as SSF	1
Mixed Human/Non-Human (n = 1)	Mixed VCA	1
**VCA- Basic Science Studies**		
Article (n = 32)	VCA Procurement/Allocation/Implementation	15
	Immunology	15
	Topic Overview	1
	VCA Monitoring Technology	1

T100 papers were cross-referenced using *WoS “Meso”* and *“Micro”* subject subheadings [Table 3]. Much of the T100 outputs were centred around the application of complex tissue flaps (n = 61) as is the cornerstone of VCA transplantation. VCA immunology, Organ Donation & Transplantation and Stem Cell Research were common *Meso* Topics also [Table 3].

**Table 3:** T100 Outputs in accordance with WoS Meso and Micro Subject Headings.

WoS *Meso* Topic	(n = )	WoS *Micro* Topic	(n = )
Cosmetic Surgery	61	Flap	61
Immunology	9	T Cell Receptor	6
		FOXP3	3
Organ Donation & Transplantation	6	Antibody-Mediated Rejection	2
		Cyclosporine	1
		Lung Transplantation	1
		Organ Donation	1
		Biliary Atresia	1
Stem Cell Research	4	Mesenchymal Stem Cells	3
		Decellularization	1
Digestive System Disorders	3	Short Bowel Syndrome	3
Immunology & Haematolog**y**	3	Complement	3
Micro & Long Noncoding RNA	1	Exomes	1
Parasitology	1	Scabies	1
Pelvic & Renal Disorders	1	Bladder Cancer	1
Sensors & Tomography	1	Electrical Impedance Tomography	1
Breast Cancer Screening	1	Lymphoedema	1
Not Listed (n = 9)			

Linear regression analysis evaluating factors affecting AAS was performed with news mentions (r = 6.95), blog mentions (r = 6.20) and patent filing mentions (r = 0.56)demonstrating the greatest positive impact on AAS (p < 0.05). With regards to social media interaction, the greatest positive predictor of AAS was twitter mentions (coefficient = 0.53 [p < 0.001]), with reddit mentions displaying a negative correlation with AAS (coefficient = −5.94 [p < 0.001]). Other social media sites did not demonstrate statistically significant correlations with AAS [Table 4].

**Table 4:** Linear regression analysis: Altmetric Attention Scores (AAS) for each outlet of dissemination for the T100 studies involving Vascularised Composite Allograft (VCA).

Altmetric Attention Score (AAS)	Coefficient	Standard Error	t	P>|t|	95 % Confidence Interval	
News Mentions	6.95	0.13	51.67	<0.001	6.67	7.22
Blog Mentions	6.20	1.15	5.39	<0.001	3.91	8.49
Patent Mentions	0.56	0.13	4.42	<0.001	0.31	0.81
Twitter Mentions	0.52	0.22	23.82	<0.001	0.48	0.57
Reddit Mentions	−5.94	1.32	−4.51	<0.001	−0.14	−3.33
Dimensions Citations	0.03	0.01	2.13	0.04	0.002	0.05
Public Policy Mentions	1.99	1.06	1.88	0.06	−0.12	4.09
Facebook Mentions	−0.82	0.16	−0.51	0.62	−0.41	0.24
Mendeley Readers (No.)	−0.02	0.01	−1.74	0.85	−0.04	0.002

**Fig. 1:**
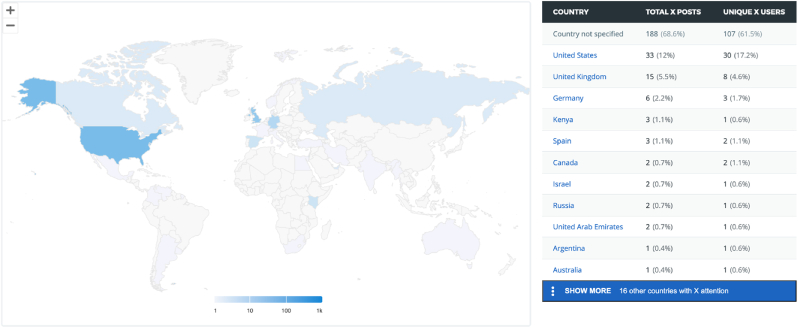
Geographical distribution of Twitter/X Mentions on the topic of VCA (Image Generated using Altmetric Explorer).

**Fig. 2:**
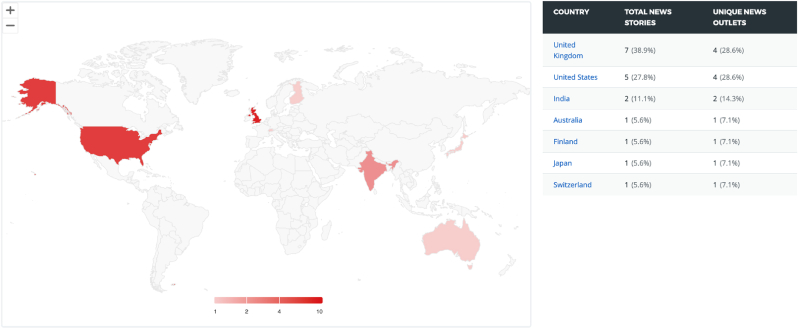
Geographical distribution of news outputs (Image Generated using Altmetric Explorer).

The geographical location of social media mentions showed a preponderance to English speaking countries with Twitter/X discussion regarding the topic of VCA being most frequently observed in The United States and The United Kingdom with 12 % and 5.5 % of total mentions respectively (Fig. 1). However, this may not be representative as the highest proportion of mentions were recorded from accounts without confirmed geographic locations (61.5 %). News stories and outlet outputs demonstrated a similar trend with the United Kingdom and United States contributing 67.7 % of total news outputs (Fig. 2).

**Fig. 3:**
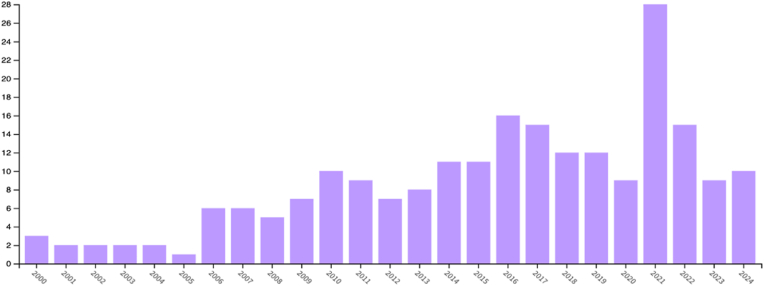
Trend in frequency of VCA research outputs from 2000 to 2024 (Graph generated using Web of Science).

**Fig. 4:**
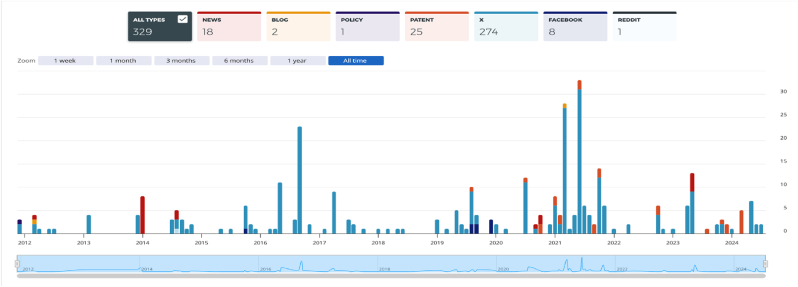
Trend in interaction surrounding the topic of VCA from 2012 to 2024 as represented by Twitter/X/Facebook Interactions, News Mentions, Blog Post Mentions, Public Policy Citations and Patent Citations (Graph generated using Altmetric Explorer).

The most featured journal in T100 publications was *Transplantation* (n = 20), followed by *American Journal of Transplantation, Plastic and Reconstructive Surgery, PRS Global Open, Transplant International* which each contributed equally (n = 7). The Ten journals which contributed to the highest number of T100 publications are outlined in Table 5. The Web of Science (WoS) database search returned 228 papers related to the topic of VCA. The frequency of publication of VCA research outputs has shown a general increase over time (Fig. 3). This generally positive trend in research outputs has been reflected also in altmetric attention as represented in Fig. 4 indicating increased interest in VCA as both an emerging area of clinical research and of public discussion.

**Table 5:** Distribution of T100 articles by Journal of Publication.

Journal	Number of T100 Publications
Transplantation	20
American Journal of Transplantation	7
Plastic and Reconstructive Surgery	7
PRS Global Open	7
Transplant International	7
Current Opinion in Organ Transplantation	4
Journal of Surgical Research	4
Vascularised Composite Allotransplantation	4
PLOS ONE	3
Annals of Plastic Surgery	2

The Top 20 performing (T20) outputs from altmetric and bibliometric databases were compared [Table 4]. Interestingly, the mean AAS was higher in the bibliometric T20 articles. The ability for WOS to generate results based on MeSH search headings likely allows for a greater capturing of the topic. The search on VCA failed to return a key paper related to penile transplantation, which was a high performer in AAS – the title and abstract did not include the term “vascular”. Journal impact factor, H-index and SJR were largely comparable. Altmetric outputs identified high performing articles in a shorter time since publication however with mean years since publication being significantly shorter at 5.80 in comparison to 10.60. Citation density was calculated as significant disparity between WOS and Google Scholar citation count has been previously identified in the literature.^24^ Unsurprisingly, WOS identified outputs with significantly higher citation density. T20 articles as identified by both WOS and Altmetric Explorer were predominantly published in open access journals [Table 6].

**Table 6:** Comparison of T20 articles on the topic of VCA from bibliometric scoring (WOS) and Altmetric scoring perspectives.

Article Characteristic		T20 WOS	T20 AAS
Journal Quartile	Q1	13	12
	Q2	4	8
	Q3	3	0
**AAS** (mean)		15.00 (0–84)	12.80 (3–60)
**Journal Impact Factor** (mean)		4.38 (0.97–13.44)	4.02 (1.33–13.44)
**H-Index** (mean)		164.20 (58–335)	165.25 (30–274)
**SJR** (mean)		1.67 (0.32–3.66)	1.77 (0.52–6.36)
**Years since Publication** (mean)		10.60 (5–21)	5.80 (1–13)
**Mendeley Readers** (mean)		60.60 (35–142)	24.65 (0–140)
**Mean Citation (WOS/Google Scholar)**		80.30 (46.5–162.5)	25.48 (0–132)
**Citation Density** (mean)		8.27 (3.37–14.77)	3.50 (0–14.66)
**Open Access (x/20)**		17	14

**Fig. 5:**
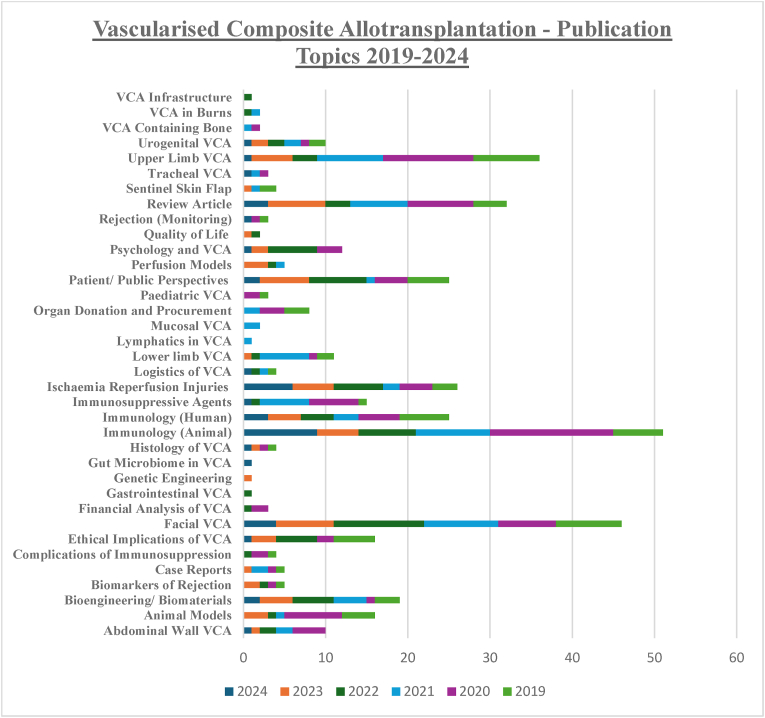
Publications relating to Vascularised Composite Allotransplantation between 2019 and 2024, which have been subdivided based on their primary focus.

Recent trends in VCA publications assessed by reviewing VCA publications returned from the WOS database over a five-year period. Two independent reviewers (CMH & GPD) screened all publications related to VCA from 2019 to 2024 (n = 413). Topic headings were assigned based on the dominant theme of the paper. In cases of disagreement, a third reviewer (GGC) provided consensus decision. Over this timeframe, the largest number of publications assessed immunological characteristics of VCA in animal models (n = 51), garnering insights from cellular, genetic and transcriptomic perspectives. The most investigated VCA model was that of facial transplantation (n = 46), with a proportion of these papers highlighting the role of mucosa in identifying rejection episodes. The evolving role of mucosa in VCA was reflected further with two stand-alone publications aimed at better characterising the macro and microscopic features of mucosa during allograft rejection. There was a significant interest in the literature in ameliorating the risk of ischaemia reperfusion injury (IRI) within the field of VCA (n = 26). The trends in publication topics have been represented graphically (Fig. 5).

## 4 Discussion

Vascularised Composite Allograft transplantation is an exciting and rapidly advancing innovative field in transplantation.^25^ The past three decades have born witness to many novel advancements from the first upper limb transplant in 1998, to the first facial allotransplantation in 2005.^26^ In the interim, >100 successful upper limb and >48 facial allotransplantations have been carried out worldwide.^27^^,^^28^ Each of these transplantations have provided essential intervention for patients with complex tissue loss not amenable to autologous tissue reconstruction with favourable functional and aesthetic outcomes. Furthermore, each of these VCA's have provided significant insight into organ transplantation from both immunological and surgical technique perspectives.^26^^,^^29^^,^^30^ The broad clinical application of VCA is impeded at present by the risk of acute and chronic allograft rejection. Publications regarding this topic over the past 5 years have reflected this challenge with advances in bioengineering strategies, mechanistic VCA immunology and novel means of monitoring VCA rejection being the top trending publications (Fig. 5). Studies investigating Facial VCA contributed to the greatest number of publications, over this five-year period, with significant interest being garnered by the role of mucosal VCA components in identifying rejection. Such publications have begun to challenge the paradigm that skin is the most immunogenic component of VCA, which may advance future clinical applications of Sentinel Skin Flap Transplantation.

So why is dissemination of VCA-related research important? Despite the forementioned advancements, the major obstacle to long term vascularised composite allograft survival and hence more widespread clinical application, is rejection. At present the incidence of rejection in VCA surpasses that of SOT and is a major focus of ongoing research.^26^^,^^29^^,^^30^ Such advancements are fundamentally reliant on the generation of research outputs, multi-institutional collaboration and sharing of globally niche data. The clinical and academic impact of such outputs are underpinned by optimal and timely dissemination of findings. Altmetrics offer a means of appraising research dissemination which is reflective of the increasingly varied means in which members of the public, medical and academic personnel alike interact with scholarly outputs.^20^

Timely and optimal dissemination of research outputs could be achieved by exploiting the modern modalities in which scholarly outputs are interacted with. Although linear regression analysis did not demonstrate a statistically significant relationship between AAS and journal quartile, Q1 journal outputs did represent the largest proportion of T100 outputs with higher median AAS. This indicates that traditional research metrics remain important with regards to research impact. The goal of publishing in higher impact journals will likely remain omnipresent among authors. Hence, when selecting journals to submit publications relating to VCA, alongside the journal quartile, clinicians should consider the journals social media presence. This study demonstrated that mean AAS was significantly higher amongst papers published in journals with dedicated and active social media sites. Early interactions with regards to a research output on Twitter, now known as “X”, has been previously shown to accurately predict an articles future citation count, with the first three days being the most influential for garnering online attention.^31^ The influence of Twitter/X was further reflected in this study which found twitter mentions to have the highest positive correlation with AAS from a social media standpoint.

Clinical research, particularly clinical trials, rely fundamentally on patient recruitment. Previous research has demonstrated the influence of social media with regards to both recruitment and increasing public awareness.^32^^,^^33^ Hence, investment in social media engagement may have benefits for researchers that reaches beyond their citation count. Such studies examining factors influencing public education and recruitment, have identified unification of voice being as an important factor in engaging members of the public.^32^ As such, we must consider the concerted message we wish to convey to members of the public if we are to optimise their understanding and willingness to engage in our research. Interestingly, only Reddit mentions demonstrated a negative directionality when regressed against AAS scores. This may potentially be attributed to the nature of the platform itself. Reddit, known for its diverse and often critical user base, can host in-depth discussions that include both positive and negative commentary. Research discussed on Reddit may be scrutinised more rigorously, leading to mixed perceptions that may affect the overall altmetric score.^34^

The importance of interaction between researchers and public policy makers has been previously investigated with this interface proving important for implication of evidence-based recommendations.^35^^,^^36^ Such studies recommend early involvement of public policy makers in research strategies to aid in generating research which will provide an evidence basis upon which meaningful policy change can be generated. Inversely, this study has demonstrated that the citation of such scholarly outputs as the basis for public policy amendments positively influences AAS.

Discussion of research outcomes on the news and on blogging websites displayed a statistically significant impact on AAS. This correlation seems sensible considering the large audience size available to news outlets as well as the trust placed in these outlets by the public. However, such streams were infrequently utilised by the T100 papers. This lack of utilisation may be underpinned by the lack of understanding amongst members of the public regarding health research concepts.^37^ If researchers want to increase public understanding and engagement in our scholarly outputs it is the responsibility of researchers to disseminate findings in language which is reasonably understandable to members of the public and policy members alike.

The limitations of Altmetrics have been previously discussed in the literature with the low proportion of articles achieving non-zero scores being highlighted as a limiting factor for statistical appraisal of outputs.^38^ The high number of zero-scored items returned on the topic of VCA likely resulted in many variables not reaching statistical significance. Furthermore, Altmetrics cannot differentiate between positive and negative online interaction which should be considered when interpreting Altmetric scores.^39^

## 5 Conclusion

Vascularised Composite Allograft Transplantation is an evolving therapeutic modality. The reach of academic outputs in VCA, as indicated by AAS, was positively impacted by Journal Quartile, publication in open access journals, news outlet mentions and various forms of interaction on social media sites such as ‘X’. Further publications in this area should maximise the academic and societal reach of their outputs by targeting such avenues. This study demonstrates that altmetric analysis provides valuable insights into the online engagement and impact of VCA transplantation research. Researchers can maximise their altmetric scores by leveraging social media and news platforms. The broader implications for VCA and future research dissemination strategies suggest that Altmetrics can complement traditional citation metrics, offering a more comprehensive overview of research impact.

## Declaration of competing interest

The authors declare that they have no known competing financial interests or personal relationships that could have appeared to influence the work reported in this paper.
