# Supplementary material for: Vascularised Composite Allotransplantation – A guide to optimal dissemination of scientific outputs
Source: J Hand Microsurg. 2024 Sep 21;17(1):100161. doi: 10.1016/j.jham.2024.100161 (PMC11770220; doi:10.1016/j.jham.2024.100161)
Supplement: Multimedia component 1 [file mmc1.docx]

| AAS | Title | Journal/Collection Title | Year of Publication | OPEN/ PAID ACCESS | Social Media Presence | Country of Journal | Discipline of Journal | SJR (1-4) | Impact Factor | Mean Citation |
| --- | --- | --- | --- | --- | --- | --- | --- | --- | --- | --- |
| 60 | Vascularized Composite Allograft Tolerance Across MHC Barriers in a Large Animal Model | American Journal of Transplantation | 2014 | OPEN | YES | United Kingdom | Transplantation | 1 | 6.38 | 82 |
| 36 | Feasibility Study of Vascularized Composite Urinary Bladder Allograft Transplantation in a Cadaver Model | The Journal of Urology | 2021 | PAID | YES | United States | Surgery | 1 | 13.44 | 132 |
| 32 | A single localized dose of enzyme-responsive hydrogel improves long-term survival of a vascularized composite allograft | Science Translational Medicine | 2014 | PAID | YES | United States | Medicine (Miscillaneous) | 1 | 6.38 | 74 |
| 25 | Robotic Vascularized Composite Bladder Allograft Transplantation | The Journal of Urology | 2022 | PAID | YES | United States | Surgery | 2 | 2.11 | 3.5 |
| 19 | Vascularized Bone Marrow-Based Immunosuppression Inhibits Rejection of Vascularized Composite Allografts in Nonhuman Primates | American Journal of Transplantation | 2011 | OPEN | YES | United Kingdom | Transplantation | 1 | 3.84 | 16.5 |
| 12 | A Single Center's Experience with Donation of Facial Allografts for Transplantation | Vascularized Composite Allotransplantation (SAGE Open Medicine) | 2016 | OPEN | NO | United Kingdom | Medicine (Miscillaneous) | 1 | 6.92 | 3.5 |
| 8 | Renal hemofiltration prevents metabolic acidosis and reduces inflammation during normothermic machine perfusion of the vascularized composite allograft: A preclinical study | Artificial Organs | 2021 | PAID | YES | United Kingdom | Medicine (Miscillaneous) | 1 | 3.84 | 10 |
| 7 | Vascularized composite allograft-specific characteristics of immune responses | Transplant International | 2016 | OPEN | YES | Italy | Transplantation | 2 | 2.21 | 2 |
| 6 | Lymphocytic Vasculitis Associated With Mild Rejection in a Vascularized Composite Allograft Recipient: A Clinicopathological Study | Transplantation | 2020 | OPEN | YES | United States | Transplantation | 2 | 2.21 | 2 |
| 6 | Targeted Complement Inhibition Protects Vascularized Composite Allografts From Acute Graft Injury and Prolongs Graft Survival When Combined With Subtherapeutic Cyclosporine A Therapy | Transplantation | 2017 | OPEN | YES | United States | Transplantation | 1 | 3.98 | 0 |
| 6 | Prevalence and Distribution of Potential Vascularized Composite Allotransplant Donors, Implications for Optimizing the Donor-recipient Match | PRS Global Open | 2018 | OPEN | YES | United States | Surgery | 1 | 6.7 | 1.5 |
| 5 | In Situ Pre-Treatment of Vascularized Composite Allografts With a Targeted Complement Inhibitor Protects Against Brain Death and Ischemia Reperfusion Induced Injuries | Frontiers in immunology | 2021 | OPEN | YES | Switzerland | Immunology | 2 | 1.33 | 0 |
| 5 | Vascularized Composite Allograft Donation and Transplantation: A Survey of Public Attitudes in the United States | American Journal of Transplantation | 2017 | OPEN | YES | United Kingdom | Transplantation | 1 | 4.67 | 8 |
| 5 | Vascularized Composite Allotransplant in the Realm of Regenerative Plastic Surgery | Mayo Clinic Proceedings | 2014 | PAID | YES | United Kingdom | Medicine (Miscillaneous) | 1 | 3.45 | 6 |
| 5 | A systematic review of immunomodulatory strategies used in skin-containing preclinical vascularized composite allotransplant models | Journal of Plastic, Reconstructive & Aesthetic Surgery (JPRAS) | 2022 | PAID | YES | United Kingdom | Surgery | 1 | 3.75 | 1 |
| 4 | Graft vasculopathy of vascularized composite allografts in humans: a literature review and retrospective study | Transplant International | 2019 | OPEN | YES | Italy | Transplantation | 1 | 4.44 | 19 |
| 4 | Vascularized Composite Allograft Transplant Survival in Miniature Swine | Transplantation | 2013 | OPEN | YES | United States | Transplantation | 1 | 3.84 | 51 |
| 4 | Vascularized composite allotransplants as a mechanistic model for allograft rejection ñ an experimental study | Transplant International | 2021 | OPEN | YES | Italy | Transplantation | 2 | 1.33 | 2 |
| 4 | Vascularized composite allotransplants as a mechanistic model for allograft rejection – an experimental study | Transplant International | 2021 | OPEN | YES | Italy | Transplantation | 1 | 1.7 | 18.5 |
| 3 | Biomaterials-based immunomodulation enhances survival of murine vascularized composite allografts | Biomaterials Science | 2023 | PAID | YES | United Kingdom | Biomedical Engineering | 1 | 3.84 | 1 |
| 3 | Normothermic Ex Situ Machine Perfusion of Vascularized Composite Allografts with Oxygen Microcarriers for 12 Hours Using Real-Time Mitochondrial Redox Quantification | Journal of Clinical Medicine | 2023 | OPEN | YES | Switzerland | Medicine (Miscillaneous) | 1 | 6.38 | 4 |
| 3 | Video Messaging to Increase Vascularized Composite Allograft Donation Willingness in United States Military Veterans | Transplantation Direct | 2022 | OPEN | NO | United States | Transplantation | 1 | 3.84 | 34.5 |
| 3 | PC33. Comparison of Ex Vivo Normothermic Preservation of Vascularized Composite Allografts in A Porcine Model: 12 vs 24 Hour Perfusion | PRS Global Open | 2023 | OPEN | YES | United States | Surgery | 1 | 2.16 | 7 |
| 3 | Implementation of Vascularized Composite Allografts in the United States: Recommendations From the ASTS VCA Ad Hoc Committee and the Executive Committee | American Journal of Transplantation | 2011 | OPEN | YES | United Kingdom | Transplantation | 1 | 3.08 | 12 |
| 3 | Vascularized composite allotransplantation: current standards and novel approaches to prevent acute rejection and chronic allograft deterioration | Transplant International | 2015 | OPEN | YES | Italy | Transplantation | 1 | 3.84 | 10.5 |
| 2 | Outcomes of Solid Organ Transplants After Simultaneous Solid Organ and Vascularized Composite Allograft Procurements | Transplantation | 2017 | OPEN | YES | United States | Transplantation | 1 | 6.38 | 16 |
| 2 | Vascularized composite allograft rejection is delayed by infusion of IFN-γ-conditioned BMSCs through upregulating PD-L1 | Cell and Tissue Research | 2019 | OPEN | YES | Germany | Histology | 1 | 3.84 | 12 |
| 2 | Tolerance to Vascularized Composite Allografts in Canine Mixed Hematopoietic Chimeras | Transplantation | 2011 | OPEN | YES | United States | Transplantation | 1 | 3.75 | 23.5 |
| 2 | Delivery of Rapamycin Using In Situ Forming Implants Promotes Immunoregulation and Vascularized Composite Allograft Survival | Scientific Reports | 2019 | OPEN | YES | United Kingdom | Multidisciplinary | 1 | 3.84 | 8.5 |
| 2 | Targeted Migration of Human Adipose-Derived Stem Cells to Secondary Lymphoid Organs Enhances Their Immunomodulatory Effect and Prolongs the Survival of Allografted Vascularized Composites | Stem Cells | 2019 | OPEN | YES | United States | Developmental Biology | 1 | 2.16 | 1.5 |
| 2 | Development of a rat forelimb vascularized composite allograft (VCA) perfusion protocol | PLOS ONE | 2023 | OPEN | YES | United States | Multidisciplinary | 1 | 2.16 | 12.5 |
| 2 | Abstract 68: Tolerance Induction of Vascularized Composite Allografts Across a Class I Barrier in Swine | PRS Global Open | 2019 | OPEN | YES | United States | Surgery | 1 | 1.75 | 44 |
| 2 | Increasing Donation Opportunities for Vascularized Composite Allografts | Progress in Transplantation | 2016 | OPEN | NO | United States | Transplantation | 1 | 3.84 | 52.5 |
| 2 | Attitudes Toward Organ, Tissue, and Vascularized Composite Allograft (VCA) Donation and Transplantation: A Survey of United States Military Veterans | Transplantation | 2020 | PAID | YES | United States | Transplantation | 1 | 1.7 | 0 |
| 2 | Induction of Tolerance of Vascularized Composite Allografts | Transplantation | 2013 | OPEN | YES | United States | Transplantation | 1 | 5.9 | 12.5 |
| 2 | Vascularized Composite Allograft Transplantation in the United States: Current State of the Field and Future Challenges | Transplantation | 2022 | OPEN | YES | United States | Transplantation | 1 | 1.7 | 11 |
| 2 | <p>Public attitudes toward vascularized composite allograft donation: a literature review</p> | Transplant Research and Risk Management | 2019 | OPEN | NO | New Zealand | Transplantation | 2 | 1.33 | 0 |
| 1 | Scabies in a bilateral hand allograft recipient: An additional mimicker of acute skin rejection in vascularized composite allotransplantation | Transplant Infectious Disease | 2017 | PAID | NO | United Kingdom | Infectious Diseases | 2 | 2.21 | 12 |
| 1 | Partial Loss of Nasal Tissue in a Facial Vascularized Composite Allograft Patient | PRS Global Open | 2020 | OPEN | YES | United States | Surgery | 2 | 1.35 | 13 |
| 1 | Functional Abdominal Wall Reconstruction Using an Innervated Abdominal Wall Vascularized Composite Tissue Allograft: A Cadaveric Study and Review of the Literature | Journal of Reconstructive Microsurgery | 2014 | PAID | YES | United States | Surgery | 1 | 3.84 | 0 |
| 1 | Dynamic Maxillary Sinus Changes of Facial Vascularized Composite Allotransplants | Plastic and Reconstructive Surgery | 2021 | PAID | YES | United States | Surgery | 1 | 1.7 | 0 |
| 1 | A Clinically Feasible Approach to Induce Delayed Tolerance in Recipients of Prior Kidney or Vascularized Composite Allotransplants | Transplantation | 2012 | PAID | YES | United States | Transplantation | 1 | 3.84 | 0 |
| 1 | Simultaneous Transplantation of Hematopoietic Stem Cells and a Vascularized Composite Allograft Leads to Tolerance | Transplantation | 2014 | OPEN | YES | United States | Transplantation | 1 | 3.75 | 18 |
| 1 | Immunomodulatory Strategies Directed Toward Tolerance of Vascularized Composite Allografts | Transplantation | 2015 | OPEN | YES | United States | Transplantation | 1 | 2.16 | 0 |
| 1 | Ex Situ Limb Perfusion System to Extend Vascularized Composite Tissue Allograft Survival in Swine | Transplantation | 2015 | PAID | YES | United States | Transplantation | 1 | 1.96 | 5 |
| 1 | The Effect of Ex Situ Perfusion in a Swine Limb Vascularized Composite Tissue Allograft on Survival up to 24 Hours | Journal of Hand Surgery - American Edition | 2016 | PAID | YES | United Kingdom | Surgery | 1 | 1.7 | 18.5 |
| 1 | The Effect of MHC Antigen Matching Between Donors and Recipients on Skin Tolerance of Vascularized Composite Allografts | American Journal of Transplantation | 2017 | OPEN | YES | United Kingdom | Transplantation | 2 | 1.33 | 0.5 |
| 1 | Immunological and inflammatory mapping of vascularized composite allograft rejection processes in a rat model | PLOS ONE | 2017 | OPEN | YES | United States | Multidisciplinary | 2 | 2.15 | 0 |
| 1 | Effect of the vascularized bone components on the survival of vascularized composite allografts | Journal of Surgical Research | 2018 | PAID | YES | United States | Surgery | 2 | 2.27 | 6.5 |
| 1 | Utility of IL-2 Complexes in Promoting the Survival of Murine Orthotopic Forelimb Vascularized Composite Allografts | Transplantation | 2018 | OPEN | YES | United States | Transplantation | 2 | 3.51 | 1 |
| 1 | Toward Development of the Delayed Tolerance Induction Protocol for Vascularized Composite Allografts in Nonhuman Primates | Plastic and Reconstructive Surgery | 2020 | PAID | YES | United States | Surgery | 2 | 1.46 | 4 |
| 1 | PERFUSION OF VASCULARIZED COMPOSITE ALLOGRAFTS WITH A TARGETED COMPLEMENT INHIBITOR PROTECTS AGAINST BRAIN DEATH INDUCED AND ISCHEMIA REPERFUSION INJURY | Transplantation | 2020 | OPEN | YES | United States | Transplantation | 3 | 1.45 | 0.5 |
| 1 | Cutaneous leukocyte lineages in tolerant large animal and immunosuppressed clinical vascularized composite allograft recipients | American Journal of Transplantation | 2021 | OPEN | YES | United Kingdom | Transplantation | 3 | 0.97 | 0 |
| 1 | Randomized preclinical study of machine perfusion in vascularized composite allografts | British Journal of Surgery | 2021 | OPEN | YES | United Kingdom | Surgery | 2 | 2.21 | 29.5 |
| 1 | Exosomes from donor‐derived adipose mesenchymal stem cells prolong the survival of vascularized composite allografts | Journal of Cellular Physiology | 2021 | PAID | YES | United States | Clinical Biochemistry | 2 | 1.33 | 9 |
| 1 | Optimization of Ex Vivo Machine Perfusion and Transplantation of Vascularized Composite Allografts | Journal of Surgical Research | 2022 | OPEN | YES | United States | Surgery | 1 | 6.38 | 44.5 |
| 1 | Continuous versus Pulsatile Flow in 24-Hour Vascularized Composite Allograft Machine Perfusion in Swine: A Pilot Study | Journal of Surgical Research | 2023 | PAID | YES | United States | Surgery | 2 | 2.21 | 26 |
| 1 | Methods of ex vivo analysis of tissue status in vascularized composite allografts | Journal of Translational Medicine | 2023 | OPEN | YES | United Kingdom | Biochemistry, Genetics and Molecular Biology | 1 | 6.38 | 33.5 |
| 1 | Local Delivery of Adipose Stem Cell Promotes Allograft Survival in A Rat Hind Limb Model of Vascularized Composite Allotransplantation | Plastic and Reconstructive Surgery | 2023 | PAID | YES | United States | Surgery | 2 | 2.21 | 103.5 |
| 1 | Infrared Fluorescence Imaging of Lymphatic Regeneration in Nonhuman Primate Facial Vascularized Composite Allografts | Annals of Plastic Surgery | 2012 | PAID | YES | United States | Surgery | 2 | 2.15 | 1 |
| 1 | Evaluation of PLGA microspheres with triple regimen on long‐term survival of vascularized composite allograft – an experimental study | Transplant International | 2020 | OPEN | YES | Italy | Transplantation | 1 | 3.84 | 2.5 |
| 1 | 123. Comparison of Ex Vivo Normothermic Preservation of Vascularized Composite Allografts in a Porcine Model: 12 vs 24 Hour Perfusion? | PRS Global Open | 2023 | OPEN | YES | United States | Surgery | 1 | 3.84 | 4.5 |
| 1 | Reconstruction of Large Abdominal Wall Defects Using Neurotized Vascular Composite Allografts | Plastic and Reconstructive Surgery | 2015 | OPEN | YES | United States | Surgery | 3 | 0.79 | 5.5 |
| 1 | OPTN Vascularized Composite Allograft Waiting List: Current Status and Trends in the United States | Transplantation | 2018 | OPEN | YES | United States | Transplantation | 3 | 0.5 | 1 |
| 1 | Regional delivery of immunosuppression for transplantation of vascularized composite allografts: opportunities near and far | Annals of Translational Medicine | 2021 | OPEN | NO | China | NONE | 3 | 3.61 | 1 |
| 1 | Allocation of Vascularized Composite Allografts | Transplantation | 2012 | PAID | YES | United States | Transplantation | 1 | 1.7 | 10 |
| 1 | Chronic Allograft Deterioration: A Clinical Reality in Vascularized Composite Allotransplantation | American Journal of Transplantation | 2017 | OPEN | YES | United Kingdom | Transplantation | 1 | 3.84 | 21 |
| 1 | Living Donation of Vascularized Composite Allografts | Plastic and Reconstructive Surgery | 2018 | PAID | YES | United States | Surgery | 2 | 1.33 | 0 |
| 1 | Large Animal Models of Vascularized Composite Allotransplantation: A Review of Immune Strategies to Improve Allograft Outcomes | Frontiers in immunology | 2021 | OPEN | YES | Switzerland | Immunology | 2 | 4.86 | 2 |
| 1 | More than skin deep: Mucosal pathology in facial vascularized composite allografts. Commentary on “Pathologies of oral and sinonasal mucosa following facial vascularized composite allotransplantation” | Journal of Plastic, Reconstructive & Aesthetic Surgery (JPRAS) | 2021 | PAID | YES | United Kingdom | Surgery | 2 | 2.19 | 8.5 |
| 1 | Donor and procurement related issues in vascularized composite allograft transplantation | Current Opinion in Organ Transplantation | 2013 | PAID | YES | United States | Transplantation | 2 | 2 | 18 |
| 1 | Chest Wall, Thymus, and Heart Vascularized Composite Allograft Proof of Concept Cadaveric Model for Heart Transplantation | Annals of Plastic Surgery | 2014 | PAID | YES | United States | Surgery | 2 | 2.19 | 25.5 |
| 1 | The potential role for regulatory T-cell therapy in vascularized composite allograft transplantation | Current Opinion in Organ Transplantation | 2014 | PAID | YES | United States | Transplantation | 2 | 2.19 | 15.5 |
| 1 | Vascularized Composite Allografts: Procurement, Allocation, and Implementation | Current Transplantation Reports | 2014 | OPEN | YES | United States | Nephrology | 2 | 1.46 | 4.5 |
| 1 | Skin Allograft and Vascularized Composite Allograft | Journal of burn care & research | 2014 | PAID | YES | United States | Emergency medicine | 1 | 3.84 | 0 |
| 1 | The landscape of vascularized composite allograft donation in the United States | Current Opinion in Organ Transplantation | 2019 | OPEN | YES | United States | Transplantation | 2 | 2.19 | 27.5 |
| 1 | Effect of Immunosuppression on the Hybrid Skeleton of Vascularized Composite Allotransplants? | PRS Global Open | 2022 | OPEN | YES | United States | Surgery | 3 | 0.79 | 6 |
| 1 | Real-Time Monitoring Using Multiplexed Multi-Electrode Bioelectrical Impedance Spectroscopy for the Stratification of Vascularized Composite Allografts: A Perspective on Predictive Analytics | Bioengineering | 2023 | OPEN | YES | Switzerland | Bioengineering | 1 | 1.46 | 2 |
| 0 | Lymphoid neogenesis in skin of human hand, nonhuman primate, and rat vascularized composite allografts | Transplant International | 2014 | OPEN | YES | Italy | Transplantation | 1 | 2.36 | 14 |
| 0 | Vascularized Composite Allograft Transplantation to Monitor Intestinal Transplant Rejection | Transplantation | 2017 | OPEN | YES | United States | Transplantation | 2 | 1.35 | 2.5 |
| 0 | Outcomes of solid organ transplants following simultaneous solid organ and vascularized composite allograft procurements: A nationwide analysis | Vascularized Composite Allotransplantation | 2016 | OPEN | NO | United Kingdom | Medicine (Miscillaneous) | 1 | 1.46 | 0 |
| 0 | Requirement for Vascularized Bone Marrow in Rejection-Free Survival of Facial Composite Tissue Allografts in Non-Human Primates. | Plastic and Reconstructive Surgery | 2010 | OPEN | YES | United States | Surgery | 2 | 2.27 | 1.5 |
| 0 | Local administration of Mitomycin‐C‐Treated peripheral blood mononuclear cells (PBMCs) prolongs allograft survival in vascularized composite allotransplantation | Microsurgery | 2015 | PAID | NO | United States | Surgery | 2 | 3.84 | 3 |
| 0 | Reconstruction of Large Abdominal Wall Defects Using Neurotized Vascular Composite Allografts | Plastic and Reconstructive Surgery | 2016 | PAID | YES | United States | Surgery | 1 | 4.03 | 25.5 |
| 0 | Mechanisms of Vascularized Bone Marrow Graft Protection of Vascularized Composite Allografts | Transplantation | 2018 | OPEN | YES | United States | Transplantation | 1 | 3.84 | 6.5 |
| 0 | Graft-implanted, enzyme responsive, tacrolimus-eluting hydrogel enables long-term survival of orthotopic porcine limb vascularized composite allografts: A proof-of-concept study | PLOS ONE | 2019 | OPEN | YES | United States | Multidisciplinary | 1 | 1.7 | 3 |
| 0 | 422.2: Investigating Galectin-3 as a Novel Therapeutic Target To Block Inflammation and Reduce Ischemia Reperfusion Injury During Vascular Composite Allograft Transplantation | Transplantation | 2022 | OPEN | YES | United States | Transplantation | 2 | 1.33 | 8 |
| 0 | 2497: Near normothermic ex-situ perfusion extends human limb allograft survival up to 24 hours | Vascularized Composite Allotransplantation | 2016 | PAID | NO | United Kingdom | Medicine (Miscillaneous) | 2 | 2.33 | 3.5 |
| 0 | Systematic pathological component scores for skin-containing vascularized composite allografts | Vascularized Composite Allotransplantation | 2017 | OPEN | NO | United Kingdom | Medicine (Miscillaneous) | 1 | 3.84 | 0 |
| 0 | Local Immunosuppression for Vascularized Composite Allografts: Application of Topical FK506-TyroSpheres in a Nonhuman Primate Model | Journal of burn care & research | 2020 | PAID | YES | United States | Emergency medicine | 2 | 2.27 | 6.5 |
| 0 | Rat Hind Limb Transplant Model to Assess Vascularized Composite Allograft (VCA) Ischemic Reperfusion Injury and Inflammation | PRS Global Open | 2020 | OPEN | YES | United States | Surgery | 2 | 2.27 | 0 |
| 0 | Vascularized Bone Marrow Cellular Depletion or Discontinuity Abrogates Protection of Vascularized Composite Allografts in Nonhuman Primates | Transplantation Direct | 2021 | OPEN | NO | United States | Transplantation | 2 | 2.21 | 29 |
| 0 | Tacrolimus-Eluting Disk within the Allograft Enables Vascularized Composite Allograft Survival with Site-Specific Immunosuppression without Systemic Toxicity | Pharmaceutical Research | 2022 | PAID | YES | United States | Biotechnology | 1 | 1.49 | 9.5 |
| 0 | In Vivo Activity of Genetically Modified Cells Preseeded in Rat Vascularized Composite Allografts | Transplantation Proceedings | 2021 | PAID | NO | United States | Transplantation | 1 | 3.84 | 41.5 |
| 0 | Tacrolimus before CTLA4Ig and rapamycin promotes vascularized composite allograft survival in MGH miniature swine | Transplant Immunology | 2022 | PAID | NO | Netherlands | Immunology | 1 | 6.38 | 4.5 |
| 0 | On Poetry and Vascularized Composite Allografting | Transplantation | 2020 | OPEN | YES | United States | Transplantation | 1 | 6.83 | 0 |
| 0 | Response Regarding: Continuous Versus Pulsatile Flow in 24-h Vascularized Composite Allograft Machine Perfusion in Swine: A Pilot Study | Journal of Surgical Research | 2023 | PAID | YES | United States | Surgery | 1 | 3.84 | 18.5 |
| 0 | Immunology of Vascularized Composite Allografts | Clinical & Developmental Immunology | 2013 | OPEN | YES | Egypt | Immunology | 1 | 6.92 | 5.5 |
| 0 | Chronic rejection in vascularized composite allografts | Current Opinion in Organ Transplantation | 2014 | PAID | YES | United States | Transplantation | 1 | 1.49 | 1.5 |
| 0 | Toward Increased Organ Procurement Organization Involvement in Vascularized Composite Allograft Donation | Progress in Transplantation | 2019 | PAID | NO | United States | Transplantation | 1 | 3.89 | 3 |

**Table 7:** *Table of T100 papers returned from Altmetric Explorer on the topic of Vascularised Composite Allotransplantation. Journal of publication, year of publication, open access article status, journal social media account, country & discipline of journal are included as well as traditional bibliometric data such as journal quartile (SJR 1-4), journal impact factor and mean citation count (average citation count according to two databases - Google Scholar and Web of Science).*
